# Application of metabolic phase-specific modified nutrition risk in critically ill score: a prospective observational study in critically ill patients

**DOI:** 10.3389/fnut.2024.1367727

**Published:** 2024-09-30

**Authors:** Geon Ho Lee, Ye Ju Kim, So Hyang Park, Sunny Park, Sung Yoon Lim, Soo An Choi

**Affiliations:** ^1^College of Pharmacy, Korea University, Sejong, Republic of Korea; ^2^College of Pharmacy and Research Institute of Pharmaceutical Sciences, Korea University, Sejong, Republic of Korea; ^3^Division of Pulmonary and Critical Care Medicine, Department of Internal Medicine, Seoul National University College of Medicine, Seoul National University Bundang Hospital, Seongnam, Republic of Korea

**Keywords:** intensive care units, metabolic phases, mNUTRIC score, nutritional adequacy, mortality

## Abstract

**Background and aims:**

The prevalence of malnutrition in intensive care units (ICU) is high and can be caused by poor intake or absorption of nutrients in the digestive track, as well as disease-related inflammation. As strong catabolism restricts nutrient supply and potentially leads to subsequent malnutrition, appropriate nutrition should be provided based on the metabolic status. However, nutritional support strategies for considering the metabolic phase are not well established. Therefore, this study aimed to establish a strategy for nutritional support in each phase by implementing a phase-specific modified Nutrition Risk in Critically Ill (mNUTRIC) score.

**Methods:**

This prospective observational study was conducted on all adult patients admitted to the medical ICU for at least 36 h at Seoul National University Bundang Hospital between September 2020 and September 2022. Patient nutrition assessment (mNUTRIC score), clinical information, and nutritional supply (calories and proteins) were measured twice, in the acute phase (measured at 2 days) and late phase (measured at 7 days). The relationship between nutritional supply and 28-day mortality was analyzed using multiple logistic regression according to the mNUTRIC score in the acute and late phases. Risk factors related to 28-day mortality were analyzed using univariate and multivariate Cox proportional hazards regressions.

**Results:**

Of the 631 patients admitted to the ICU during the study period, 613 were included in the acute phase and 361 patients were included in the late phase. Nutritional supply was associated with 28-day mortality, with high mNUTRIC scores in both the acute and late phases. Cox proportional hazards regression analysis demonstrated that a high mNUTRIC score [hazard ratio (HR) 3.20 and 2.52, respectively], lactate >2.5 mg/dL were independent risk factors in both the acute and late phases. In addition, Albumin <2.5 mg/dL, the presence of neoplasm, and the need for dialysis in the acute phase, calorie adequacy <0.7 in the late phase (HR, 2.19) were identified as additional risk factors.

**Conclusion:**

The mNUTRIC score is a suitable tool for identifying critically ill patients who benefit from nutritional support. Nutritional supply should be considered for patients with high mNUTRIC scores in both the acute and late phases; however, careful supply should be provided in the acute phase and sufficient supply should be provided in the late phase.

## 1 Introduction

The prevalence of malnutrition in intensive care units (ICU) is higher (38–78%) (1) than in all inpatients (30–55%) (2). Moreover, malnutrition in critically ill patients is associated with poor clinical outcomes (3), which can be improved with appropriate supply of nutrition (4, 5). However, no gold standard exists for the nutritional screening or assessment of critically ill patients (6). The application of several traditional nutrition screening or assessment tools has been validated in the ICU (1). However, the aforementioned tools appear to be inappropriate for patients due to the unavailability of subjective information and were not developed for critically ill patients (7, 8). Accordingly, a new perspective of the nutrition assessment tool called the modified Nutrition Risk in Critically Ill (mNUTRIC) score (9, 10), which is based on objective patient information in the ICU, has been proposed, and the ability of this tool to predict prognosis or identify patients who benefit from nutritional supply has been validated (10–14).

Malnutrition can be caused by poor intake or absorption of nutrients in the digestive track but can also be a result of disease-related inflammation (15, 16). In critically ill patients, as their metabolic status changes over time, the acute phase is divided into early and late periods (characterized by an increase and decrease in catabolism, respectively), and the subsequent period is defined as the late phase (post-acute phase) (6). These changes in metabolic state are directly related to nutritional requirements and the effect of external nutritional supply on patient outcomes (17). As strong catabolism in the acute phase restricts nutritional supply and potentially leads to subsequent malnutrition, appropriate nutrition should be supplied in accordance with the patient’s metabolic status at the right time (18). Therefore, nutritional status should be assessed routinely (19) and should reflect the metabolic status, especially in the ICU. However, despite strong recommendations for nutrition assessment within 48 h after admission and early nutrition for patients with malnutrition (16, 20), and the need for nutritional support strategies according to metabolic status (21), the necessity or practicality of subsequent reassessment or nutrition support strategies applying the assessments are not well established, and related studies are insufficient.

The reasons for applying the mNUTRIC score as a nutritional assessment in this study were as follows: (1) The mNUTRIC score is more objective and easier to assess than other nutritional assessments (8, 15, 22). (2) The nutrition assessment currently used to evaluate malnutrition have mainly focused on verifying prognosis predictions and have not been able to select groups of ICU patients who would actually benefit from nutritional support (7, 8, 23, 24). (3) As these tools only include long-term nutritional records, which are not associated with metabolic status after ICU admission, little difference is observed in nutritional assessment between the acute and late phases (13). We hypothesized that the mNUTRIC score, which reflects the severity of a patient’s condition, would identify patients who need nutritional support in each phase. The study aimed to validate the mNUTRIC score not only in the acute phase but also in the late phase and to establish an optimal nutritional support strategy using phase-specific assessment.

## 2 Methods

### 2.1 Study design and patient selection

This prospective observational study included all adult patients (aged 18 years and older) admitted to the medical ICU at Seoul National University Bundang Hospital between September 2020 and September 2022. Patients with less than 36 h of hospitalization in the ICU (due to death or discharge) or insufficient clinical data to conduct nutritional assessment were excluded. All data collection and analysis procedures were approved by the Institutional Review Board of Seoul National University Bundang Hospital (No. B-2009-634-301).

### 2.2 Nutritional assessment and data collection

Clinical data and basic information of the patients were collected from electronic medical records as follows: age, sex, weight, body mass index (BMI), acute physiology and chronic health evaluation II (APACHE II), sequential organ failure assessment (SOFA), comorbidities, days from hospital to ICU, use of renal replacement therapy, drugs (vasopressors, antibiotics), nutrition administration route, source of ICU admission, diagnosis in ICU, laboratory data [albumin, C-reactive protein (CRP), lactate, white blood cell (WBC), and lymphocyte]. The patients were classified as having a high score (5–9 points) or a low score (0–4 points) according to their mNUTRIC score (10). The mNUTRIC score and all clinical information were measured twice in the acute phase (measured on day 2, 36–48 h, when catabolism is expected to be highest) and late phase (measured on day 7, 156–168 h, when catabolism is expected to be most stable and just before anabolism is expected to begin) to reflect metabolic changes. The 28-day mortality rate was analyzed for all patients enrolled in this study. Based on the AUC results derived from the meta-analysis on the accuracy of the GLIM criteria (25) and the validity study for the mNUTRIC score (26) (0.82 and 0.693, respectively), the sample size is calculated as 0.05 for type 1 error, 0.8 for type 2 error, and 11 for DOR, resulting in 564 people. Considering exclusion and dropout, 10% more patient groups were recruited.

The total nutritional supply (calories and proteins) included both parenteral nutrition (PN) and enteral nutrition (EN) on days 2 and 7, respectively. Calories included all supplies, such as 5% dextrose and propofol which are not typically used for nutritional purposes. Calories and proteins supplied by EN and PN were calculated by multiplying the given volume with the calories and protein per 1 mL of each product. Calorie adequacy was calculated as total calorie supply (kcal)/calorie requirement (kcal) [calorie requirement was calculated as body weight (kg) × 25 kcal/kg]. Protein supply was calculated as the total protein intake (g)/body weight (kg). When deriving calorie requirement and protein supply, body weight was defined as dry weight, and in the case of obese patients (BMI >25) (27), the weight was defined as adjusted body weight [(actual body weight-ideal body weight) × 0.33 + ideal body weight] (6). The cut-off for low-calorie supply was set as calorie adequacy <0.5 or 0.7 considering the phases, and the cut-off of low protein supply was set as <1 g/kg/d or 1.3 g/kg/d considering that the patient group compromised old individuals (6, 28, 29) in statistical analysis. This observational study did not involve any interventions in data collection, measurement, or nutritional supply.

### 2.3 Statistical analysis

The patient characteristics were expressed as average ± standard deviation (median, interquartile range if necessary) for continuous data, and as numbers (percentages) for categorical data. The differences between groups were compared using the Student’s *t*-test for continuous data and the chi-square test for categorical data. Normality test was performed on continuous data.

The relationship between nutritional supply (calorie adequacy and protein supply) and 28-day mortality was analyzed using multiple logistic regression according to the mNUTRIC score in the acute and late phases. Risk factors related to the 28-day mortality were analyzed using univariate and multivariate Cox proportional hazards regressions in each phase. After performing a univariate analysis of the demographic factors for each phase, a multivariate regression analysis was conducted with backward elimination, including only the factors satisfying *p* < 0.1 in the univariate analysis. A multicollinearity test was performed on the included factors, and the criteria for the mNUTRIC score were not included in the multivariate regression analysis in consideration of collinearity (9). Spearman’s correlation analysis was performed to confirm the correlation between patient severity (APACHE II) and nutritional supply, demonstrating that nutritional supply was not determined by severity. All statistical analyses were two-sided tests, and a *p*-value <0.05 was considered statistically significant. Statistical analyses were performed using SAS software version 9.4.

## 3 Results

**Figure 1:**
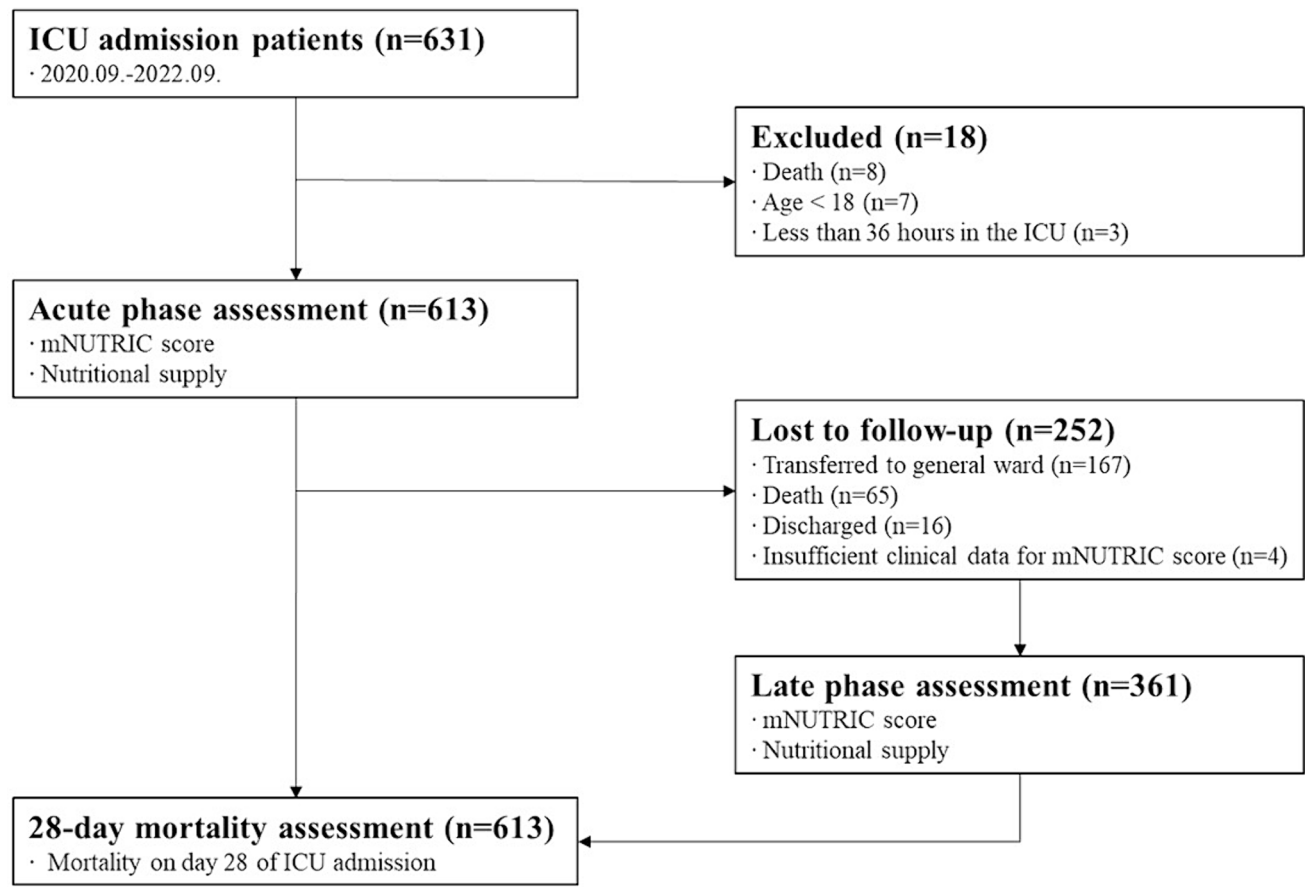
Flow diagram.

Of the 631 patients admitted to the ICU during the study period, 613 were in the acute phase. Moreover, 361 people were included in the late phase. A flow diagram is displayed in Figure 1.

Table 1 displays the baseline characteristics of patients in the acute and late phases. The mean age was 67.2 ± 15.3, and males accounted for 391 (63.78%). In the acute phase, the proportion of patients with high mNUTRIC and APACHE II/SOFA scores was significantly higher than that in the late phase. In addition, given that some laboratory parameters (albumin, CRP, lactate, and WBC) and the proportion of patients using vasopressors were also higher in the acute phase compared to the late phase, patients’ metabolic status appeared to be unstable in the acute phase. On the other hand, in the late phase, both calories and proteins were supplied more compared to the acute phase (calorie adequacy: 0.41 ± 0.36 in acute phase and 0.74 ± 0.41 in late phase; protein supply: 0.35 ± 0.41 g/kg in acute phase and 0.75 ± 0.47 g/kg in late phase). Furthermore, the proportion of EN was higher in the late phase than in the acute phase. In the late phase, although the severity decreased and nutritional supply increased, the proportion of patients with high mNUTRIC scores (53.74%) and protein supply <1 g/kg (70.64%) remained high. No significant differences in age, sex, phenotypic status (BMI and weight), or 28-day mortality between the acute and late phases were observed.

**Table 1:** Patient characteristics according to the metabolic phases.

	Acute phase (*N* = 613)	Late phase (*N* = 361)	*p*-value
mNUTRIC			<0.001
High score	485 (79.12%)	194 (53.74%)	
Low score	128 (20.88%)	167 (46.26%)	
Age (years)*	67.2 ± 15.3	67.5 ± 15.2	0.749
APACHE II score*	28.6 ± 8.7 (29, 23–35)	18.4 ± 8.3 (17, 12–24)	<0.001
SOFA score*	7.5 ± 3.5 (7, 5–10)	6.7 ± 3.5 (6, 4–9)	0.002
Comorbidities ≥2 (N, %)*	507 (82.71%)	307 (85.04%)	0.343
Days from hospital to ICU (days)*	5.6 ± 13.3 (1, 0–5)	7.1 ± 15.3 (6, 4–9)	0.132
Sex (N, %)			0.680
Male	391 (63.78%)	235 (65.10%)	
Female	222 (36.22%)	126 (34.90%)	
Weight at ICU admission (kg)	61.9 ± 14.6	62.4 ± 16.2	0.600
BMI (kg/m^2^)	23.4 ± 5.3	23.6 ± 6.0	0.543
Vasopressors (N, %)	427 (69.66%)	187 (51.8%)	<0.001
Renal dialysis (N, %)	159 (25.94%)	87 (24.10%)	0.524
Antibiotics (N, %)	521 (84.99%)	293 (81.16%)	0.119
Route of administration			<0.001
NPO	35 (5.71%)	5 (1.39%)	
EN	99 (16.15%)	89 (24.65%)	
PN	301 (49.10%)	75 (20.78%)	
EN + PN	178 (29.04%)	192 (53.19%)	
Total calorie (kcal)	589 ± 506 (448, 185–844)	1,059 ± 580 (1,079, 618–1,381)	<0.001
Calorie adequacy**	0.41 ± 0.36 (0.32, 0.14–0.60)	0.74 ± 0.41 (0.77, 0.44–1.00)	<0.001
<50% (N, %)	409 (66.72%)	109 (30.19%)	<0.001
<70% (N, %)	501 (81.73%)	164 (45.43%)	<0.001
Protein supply (g/kg)	0.35 ± 0.41 (0.23, 0–0.57)	0.75 ± 0.47 (0.75, 0.40–1.06)	<0.001
< 1.0 g/kg (N, %)	564 (92.01%)	255 (70.64%)	<0.001
< 1.3 g/kg (N, %)	593 (96.74%)	317 (87.81%)	<0.001
Diagnosis at ICU admission			0.708
Respiratory system	147 (23.98%)	93 (25.76%)	
Circulatory system	143 (23.33%)	90 (24.93%)	
Neoplasm	102 (16.64%)	52 (14.40%)	
Digestive system	44 (7.18%)	21 (5.82%)	
Infectious (including COVID-19)	44 (7.18%)	32 (8.86%)	
Others	133 (21.7%)	73 (20.22%)	
Albumin (mg/dL)	2.9 ± 0.9 (2.8, 2.4–3.2)	2.7 ± 0.4 (2.7, 2.5–2.9)	0.001
CRP (mg/L)	12.0 ± 9.3 (9.5, 4.3–18.9)	8.9 ± 7.5 (6.4, 3.5–11.6)	<0.001
Lactate (mg/dL)	3.4 ± 4.2 (2.0, 1.3–3.4)	2.2 ± 2.7 (1.5, 1.1–2.2)	<0.001
WBC (/mm^3^)	13.2 ± 10.6 (10.7, 7.7–15.8)	11.9 ± 7.5 (10.3, 7.5–14.6)	0.029
Lymphocytes (%)	9.8 ± 9.3 (7.7, 4.6–11.9)	10.7 ± 9.8 (8.5, 4.8–13.3)	0.145
28-day mortality			0.489
Death (N, %)	153 (24.96%)	83 (22.99%)	

*These are the criteria for the mNUTRIC score.

**Calorie adequacy is calculated by dividing total calorie supply by calorie requirements.

Data were partially missing for albumin (1), CRP (17), lactate (52), and lymphocytes (3) in the acute phase and albumin (3), CRP (11), lactate (59), WBC (1), and lymphocytes (3) in the late phase. ICU, intensive care unit; BMI, body mass index; NPO, Nothing per oral; EN, Enteral Nutrition; PN, Parenteral Nutrition; CRP, C-reactive protein; WBC, white blood cells.

**Figure 2:**
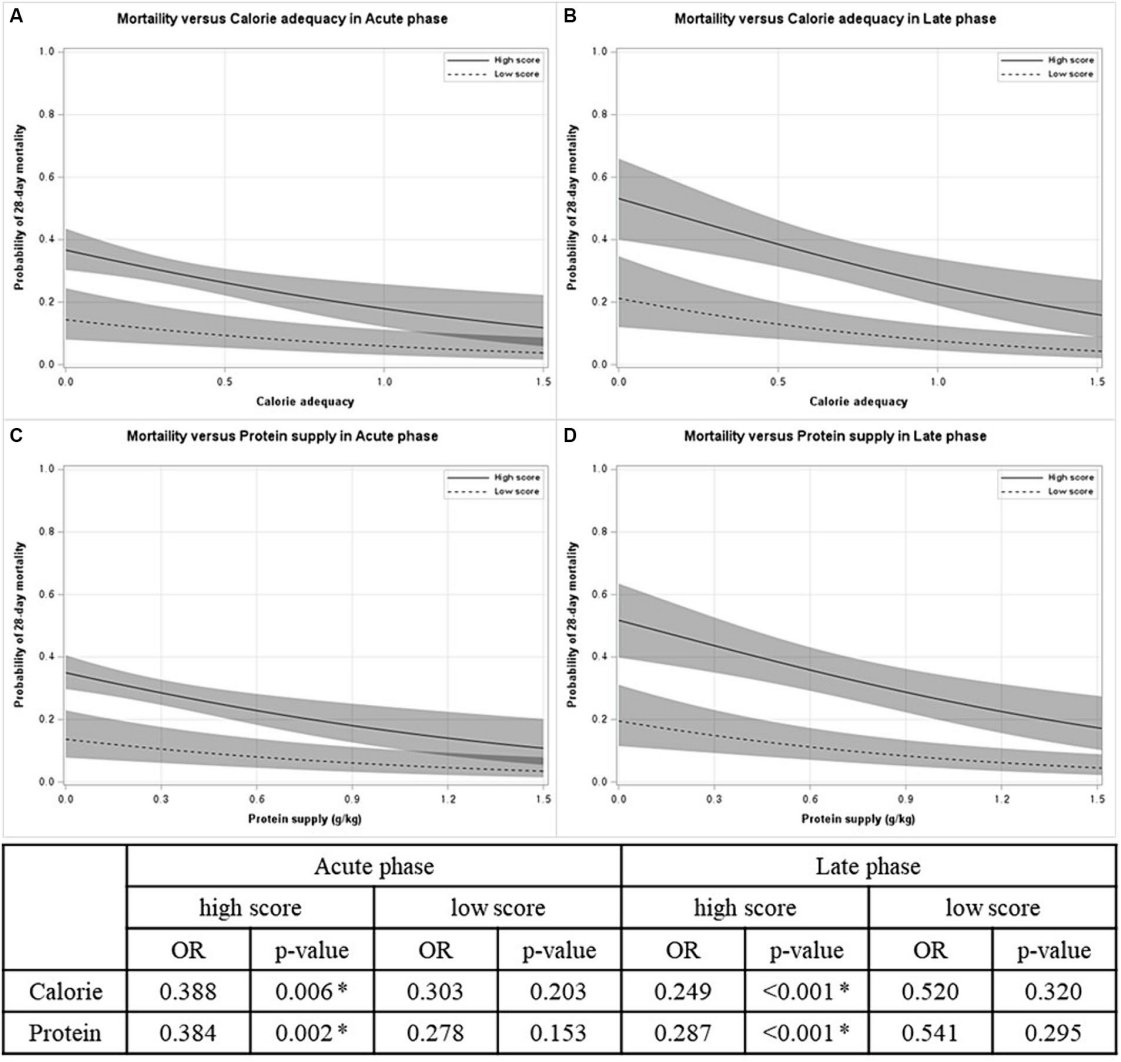
Probability of 28-day mortality versus nutritional supply by mNUTRIC score in each phase. **(A)** Mortality versus calorie adequacy in acute phase. **(B)** Mortality versus calorie adequacy in late phase. **(C)** Mortality versus protein supply in acute phase. **(D)** Mortality versus protein supply in late phase. OR odds ratio. *p < 0.05.

Figure 2 displays that nutritional supply is associated with 28-day mortality in patients with high mNUTRIC scores. In both the acute and late phases, the predicted 28-day mortality tended to decrease as the nutritional supply increased [odds ratio (OR) for calories: 0.388 in the acute phase, 0.249 in the late phase; OR for protein: 0.384 in the acute phase, 0.287 in the late phase]. On the other hand, in patients with low mNUTRIC scores, no significant association between nutritional supply and 28-day mortality, regardless of the phase exists.

**Figure 3:**
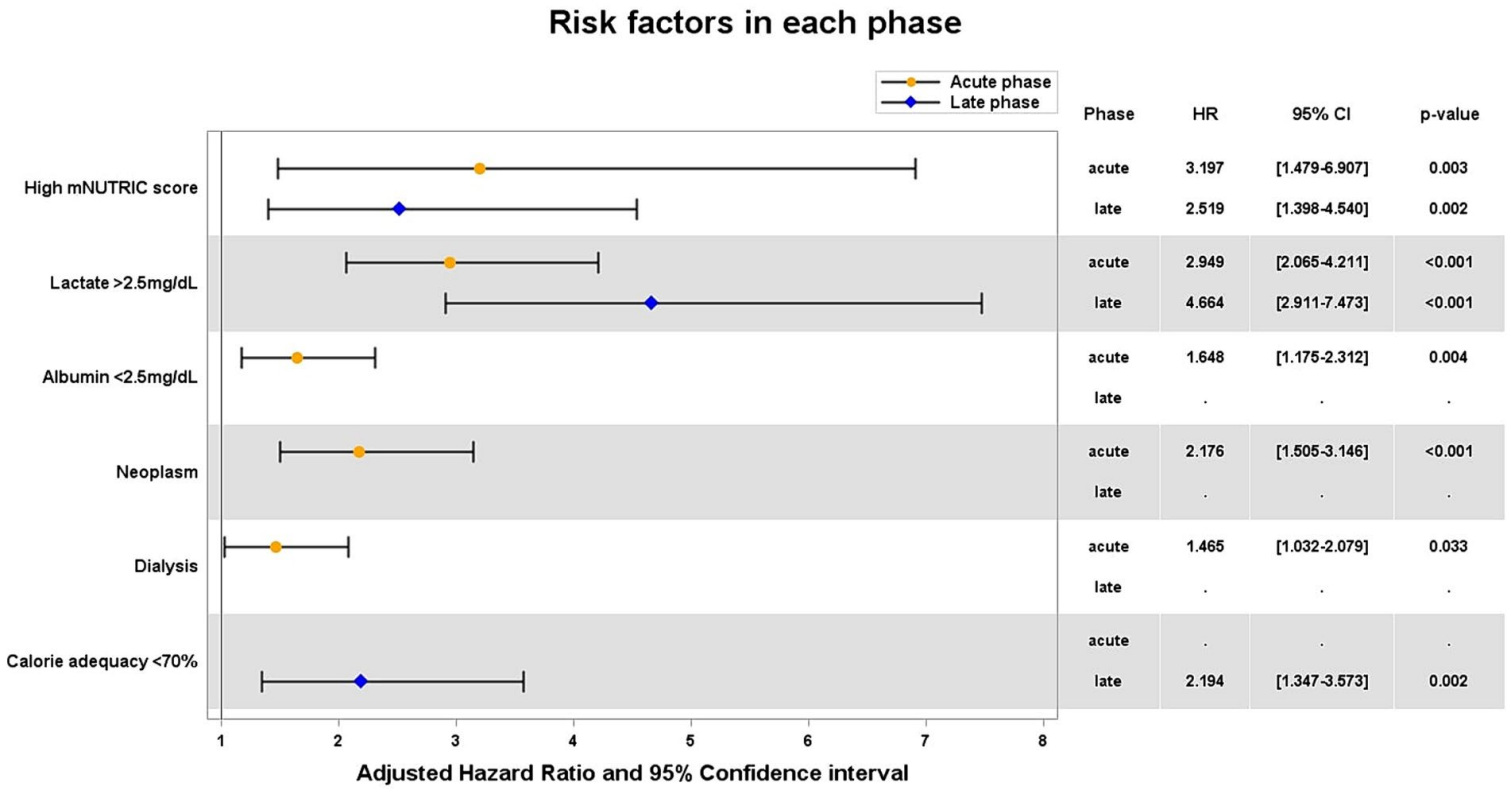
Adjusted hazard ratios of significant variables for 28-day mortality. HR hazard ratio; CI confidential interval.

As the result of Cox proportional hazards regression (Figure 3), in both acute and late phases, high mNUTRIC score [hazard ratio (HR) 3.20 and 2.52, respectively], lactate >2.5 mg/dL (HR 2.95 and 4.66, respectively) were independent risk factors that increase 28-day mortality. Albumin <2.5 mg/dL, the presence of neoplasm, and the need for dialysis were identified as additional risk factors in the acute phase. Furthermore, calorie adequacy <0.7 was identified in the late phase (HR 2.19).

## 4 Discussion

We attempted to examine the application of phase-specific mNUTRIC score, considering the metabolic status in critically ill patients. Furthermore, our findings confirmed that the mNUTRIC score can identify patient groups that benefit from nutritional supply, especially in the late phase when the metabolic status is stabilized. In addition, calorie adequacy <0.7 was identified as a risk factor for the 28-day mortality in the late phase. The strength of this study is the collection of a phase-specific nutritional assessment and nutritional supply in each phase, considering metabolic status which has not been done before.

### 4.1 Necessity and practicality of phase-specific mNUTRIC score in critically ill patients

In this study, it was confirmed that appropriate nutritional supply lowers 28-day mortality in patients with high mNUTIC scores, not only in the acute phase but also in the late phase. Therefore, the findings imply that the mNUTRIC score can identify patients who benefit from nutritional supply in both the acute and late phases. Previous similar studies have also confirmed the association between nutritional supply and patient outcomes in patients with high mNUTRIC scores (9, 10, 14, 30), but the assessment was performed only once after admission to the ICU.

Contrary to the developmental intention of the mNUTRIC score, some argue that this scoring system identifies more severely ill patients who may have reduced tolerance for increased nutrition, rather than patients who genuinely benefit from it. Since most mNUTRIC score validation studies are observational, a greater nutritional supply may have resulted in an improved prognosis (less severe), and vice versa. Therefore, some mNUTRIC validation studies demonstrate no correlation between disease severity (mNUTRIC or APACHE II scores) and nutritional supply (10, 31). However, the nutritional supply was the average value of a certain period [12 days in a study by Rahman et al. (10) and 28 days in that by Chada et al. (31)], and the score was measured only in the acute phase, which does not reflect all the conditions of that period. In our study, nutritional supply was correlated with the APACHE II score in the acute phase high-score group (*p* < 0.001 for both calories and proteins) but not in the late phase high-score group (*p* = 0.2397 and 0.1914 for calories and proteins, respectively). Furthermore, the difference in median calorie adequacy values between the high and low-score groups is more substantial in the acute phase (0.27 vs. 0.51) when compared to the late phase (0.73 vs. 0.80) (Supplementary Table S1). Therefore, identifying patients who benefit from nutritional supply is necessary along with providing appropriate nutritional supply through additional mNUTRIC scores in the late phase, when the patient’s condition stabilizes and calories can be properly supplied regardless of severity.

### 4.2 Risk factors for 28-day mortality in acute and late phases

In a variety of patient populations (including patients with Coronavirus disease 2019), groups with high mNUTRIC scores assessed after ICU admission have already been proven to have high 28-day mortality (14, 32, 33). Our previous study also demonstrated that the mNUTRIC score is superior to other nutritional assessments in terms of prognostic prediction and that prognostic prediction is more accurate in the late phase than in the acute phase (13). Moreover, in an observational study (34), a high mNUTRIC score was identified to be strongly associated with 28-day mortality (HR 4.21, [95% confidence interval: 2.70–6.58]), and other prognostic outcomes. We confirmed that a high mNUTRIC score is a factor that increases the 28-day mortality, even in the late phase.

Calorie adequacy <0.7 was observed as an additional risk factor only in the late phase. One study also discovered that a supply of more than 70% of the calorie requirement was helpful for 60-day survival (29), and the mean calorie adequacy in this study was 89%. In our study, observing the effect of calories on the 28-day mortality in the acute phase may have been difficult because calorie adequacy in the acute phase was notably low, with a mean of 41% (74% in the late phase). Additionally, in the acute phase in critically ill patients, an intrinsic energy source is produced due to strong catabolism; therefore, calorie supply may not improve patient outcomes due to metabolic overload or ubiquitin-proteasome pathway inhibition, which is important for cell repair and organ recovery (35, 36). However, as the patient’s metabolic state stabilizes in the late phase, internally produced calories decrease, and insufficient nutritional supply affects patient outcomes more negatively in this phase than in the acute phase. Therefore, more attention should be paid to nutritional supply in the late phase than in the acute phase.

Recent studies have reported that protein supply is helpful for patient outcomes in the acute phase (5, 37, 38) but other studies suggest the opposite (39, 40). In our study, protein supply was associated with 28-day mortality in patients with high mNUTRIC scores, but a protein supply of <1 or 1.3 g/kg was not identified as a risk or beneficial factor for 28-day mortality. This is probably because the protein supply in our study (mean: 0.35 g/kg in acute phase) was much lower than that in other studies [the means of each protein supply were 0.71, 0.97, 0.73 g/kg, respectively (5, 37, 38)], and therefore it was not enough to assess the risks of high protein intake [cut-off of high-protein: >0.8, 2.2 g/kg (39, 40)]. In future studies, it will be necessary to verify the effect of protein supply on patient outcome, and to define the optimal target dose.

### 4.3 Phase-specific mNUTRIC score in ICU

This study observed that a high mNUTRIC score is an independent risk factor for 28-day mortality and that nutritional supply can reduce mortality in the high-score group. Hence, identifying patients who require early nutritional intervention using the mNUTRIC score in the acute phase is feasible (16). However, the European Society for Clinical Nutrition and Metabolism (ESPEN) guidelines recommend careful monitoring of calorie supply to prevent overfeeding during the initial 48 h (acute phase) (6). Studies that caution against overlooking the potential risks associated with nutritional supply in patients with low mNUTRIC scores are also available (41, 42). In addition, a meta-analysis indicated that the effect of nutritional supply might have been overestimated (43). In our study, the effect of nutritional supply in the acute phase might be attributed to patient severity, and the benefits of nutritional supply was not identified with a low mNUTRIC score. Therefore, early nutrition should be considered in patients with high mNUTRIC scores; however, considering each patient’s condition and avoiding overfeeding by providing low calories are essential (37, 44).

In contrast, the mNUTRIC score in the late phase identified patients who required nutritional support more than those in the acute phase. According to the ESPEN guidelines (6), calorie requirements should be calculated using an indirect calorimeter; however, routine use of the calorimeter in the ICU can be challenging. Therefore, calorie requirements are generally calculated based on weight, and it is recommended not to exceed 70% of the requirement for a week. However, according to our study, less than 70% requirement is a risk factor for 28-day mortality, and the prevalence of a high mNUTRIC score remains notably high, even in the late phase (53.74%). Additionally, a large number of patients with a low mNUTRIC score in the acute phase were identified to have a high mNUTRIC score in the late phase (42). Therefore, after subdividing patients using the mNUTRIC score in the late phase, more adequate nutritional support should be provided to those who benefit from the nutritional supply.

### 4.4 Limitations

This study had certain limitations. First, a causal relationship cannot be identified in an observational study. Second, this study was conducted at a single center in South Korea. Third, the changes in metabolic status are different for each patient; therefore, the 2^nd^ and 7^th^ days we assumed may not be the acute and late phases for certain patients. Fourth, heterogeneity may be present by including all patients with a variety of diseases. Additionally, only macronutrients were included in this study. Finally, mortality may not be the best indicator of nutritional outcomes (45). Further research is necessary to analyze the effects of nutritional supply on other outcomes.

## 5 Conclusion

We demonstrated the applicability of phase-specific mNUTRIC score in the ICU. Although the mNUTRIC score does not include nutritional parameters, the score appears to be a suitable tool for identifying critically ill patients who benefit from nutrition by considering their metabolic status. In the acute phase, nutritional supply should be considered for high mNUTRIC scores reflecting each patient’s condition, whereas, in the late phase, sufficient nutrition should be provided for high mNUTRIC scores.

## Data Availability

The original contributions presented in the study are included in the article/Supplementary material, further inquiries can be directed to the corresponding authors.
