# Supplementary material for: Application of metabolic phase-specific modified nutrition risk in critically ill score: a prospective observational study in critically ill patients
Source: Front Nutr. 2024 Sep 30;11:1367727. doi: 10.3389/fnut.2024.1367727 (PMC11471547; doi:10.3389/fnut.2024.1367727)
Supplement: Supplementary file 1 [file Table_1.DOCX]

Supplementary Material

# Supplementary Tables

Table S1. Patient characteristics according to the mNUTRIC malnutrition risk by metabolic phase.

|  | **Nutrition risk assessment by mNUTRIC score in acute phase (N=613)** | | | | **Nutrition risk assessment by mNUTRIC score in late phase (N=361)** | | | | **p-value** |
| --- | --- | --- | --- | --- | --- | --- | --- | --- | --- |
|  | **Total(N=613)** | **High Score**  **(N=485, 79.12%)** | **Low Score**  **(N=128, 20.88%)** | **p-value** | **Total(N=361)** | **High Score**  **(N=194, 53.74%)** | **Low Score**  **(N=167, 46.26%)** | **p-value** | <0.001 |
| Age (years) | 67.2 ± 15.3 | 70.2 ± 13.2 | 55.5 ± 17.3 | <0.001 | 67.5 ± 15.2 | 72.2 ± 12.4 | 62.0 ± 16.3 | <0.001 | 0.749 |
| APACHE II score | 28.6 ± 8.7  (29, 23–35) | 31.2 ± 7.1  (31, 26–36) | 18.7 ± 6.4  (17, 14–23) | <0.001 | 18.4 ± 8.3  (17, 12–24) | 23.6 ± 7.0  (23, 18–28) | 12.4 ± 5.2  (12, 9–15) | <0.001 | <0.001 |
| SOFA score | 7.5 ± 3.5 (7, 5–10) | 8.3 ± 3.2 (8, 6–10) | 4.4 ± 3.0 (4,2–6) | <0.001 | 6.7 ± 3.5 6, 4–9) | 8.2 ± 3.3 (8, 6–11) | 5.1 ± 3.0 (5, 3–7) | <0.001 | 0.002 |
| Comorbidities ≥ 2 (N, %) | 507 (82.71%) | 435 (89.69%) | 72 (56.25%) | <0.001 | 307 (85.04%) | 181 (93.3%) | 126 (75.45%) | <0.001 | 0.343 |
| Days from hospital to ICU (days) | 5.6 ± 13.3 (1, 0–5) | 6.1 ± 13.3 (1, 0–6) | 3.8 ± 13.1 (0, 0–1) | 0.091 | 7.1 ± 15.3 (6, 4–9) | 8.7 ± 16.6 (2, 0–10) | 5.1 ± 13.5 (0, 0–3) | 0.023 | 0.132 |
| Sex (N, %) |  |  |  | 0.585 |  |  |  | 0.776 | 0.680 |
| Male | 391 (63.78%) | 312 (64.33%) | 79 (61.72%) |  | 235 (65.10%) | 125 (64.43%) | 110 (65.87%) |  |  |
| Female | 222 (36.22%) | 173 (35.67%) | 49 (38.28%) |  | 126 (34.90%) | 69 (35.57%) | 57 (34.13%) |  |  |
| Weight at ICU admission (kg) | 61.9 ± 14.6 | 61.7 ± 14.4 | 62.4 ± 15.4 | 0.663 | 62.4 ± 16.2 | 60.1 ± 12.5 | 65.0 ± 19.4 | 0.005 | 0.600 |
| BMI (kg/m^2^) | 23.4 ± 5.3 | 23.4 ± 5.4 | 23.1 ± 5.4 | 0.464 | 23.6 ± 6.0 | 23.1 ± 5.2 | 24.2 ± 6.8 | 0.076 | 0.543 |
| Vasopressors (N, %) | 427 (69.66%) | 370 (76.29%) | 57 (44.53%) | <0.001 | 187 (51.8%) | 119 (61.34%) | 68 (40.72%) | <0.001 | <0.001 |
| Renal dialysis (N, %) | 159 (25.94%) | 145 (29.90%) | 14 (10.94%) | <0.001 | 87 (24.10%) | 57 (29.38%) | 30 (17.96%) | 0.011 | 0.524 |
| Antibiotics (N, %) | 521 (84.99%) | 430 (88.66%) | 91 (71.09%) | <0.001 | 293 (81.16%) | 163 (84.02%) | 130 (77.84%) | 0.135 | 0.119 |
| Route of administration |  |  |  | <0.001 |  |  |  | 0.001 | <0.001 |
| NPO | 35 (5.71%) | 29 (5.98%) | 6 (4.69%) |  | 5 (1.39%) | 4 (2.06%) | 1 (0.60%) |  |  |
| EN | 99 (16.15%) | 63 (12.99%) | 36 (28.13%) |  | 89 (24.65%) | 34 (17.53%) | 55 (32.93%) |  |  |
| PN | 301 (49.10%) | 261 (53.81%) | 40 (31.25%) |  | 75 (20.78%) | 51 (26.29%) | 24 (14.37%) |  |  |
| EN+PN | 178 (29.04%) | 132 (27.22%) | 46 (35.94%) |  | 192 (53.19%) | 105 (54.12%) | 87 (52.10%) |  |  |
| Total calorie (kcal) | 588.5 ± 505.8  (447.9, 184.8–844.1) | 533.3 ± 459.9  (376.3, 184.8–766.9) | 797.5 ± 609.1  (674.2, 369.6–1032.8) | <0.001 | 1058.5 ± 580.3  (1078.8, 618.0–1380.8) | 963.2 ± 548.7  (1021.2, 520.5–1347.3) | 1169.3 ± 597.7  (1140.0, 774.0–1485.9) | 0.001 | <0.001 |
| Calorie adequacy* | 0.41 ± 0.36  (0.32, 0.14–0.60) | 0.38 ± 0.33  (0.27, 0.12–0.56) | 0.56 ± 0.44  (0.51, 0.25–0.73) | <0.001 | 0.74 ± 0.41  (0.77, 0.44–1.00) | 0.69 ± 0.40  (0.73,0.38–0.96) | 0.80 ± 0.41  (0.80, 0.51–1.09) | 0.011 | <0.001 |
| <50% (N, %) | 409 (66.72%) | 346 (71.34%) | 63 (49.22%) | <0.001 | 109 (30.19%) | 69 (35.57%) | 40 (23.95%) | 0.017 | <0.001 |
| <70% (N, %) | 501 (81.73%) | 407 (83.92%) | 94 (73.44%) | 0.006 | 164 (45.43%) | 94 (48.45%) | 70 (41.92%) | 0.214 | <0.001 |
| Protein Supply (g/kg) | 0.35 ± 0.41  (0.23, 0–0.57) | 0.30 ± 0.38  (0.14, 0–0.51) | 0.51 ± 0.48  (0.43, 0.12–0.71) | <0.001 | 0.75 ± 0.47  (0.75, 0.40–1.06) | 0.71 ± 0.48  (0.72, 0.31–1.03) | 0.78 ± 0.46  (0.78, 0.50–1.08) | 0.161 | <0.001 |
| < 1.0g/kg (N, %) | 564 (92.01%) | 453 (93.40%) | 111 (86.72%) | 0.013 | 255 (70.64%) | 141 (72.68%) | 114 (68.26%) | 0.358 | <0.001 |
| < 1.3g/kg (N, %) | 593 (96.74%) | 474 (97.73%) | 119 (92.97%) | 0.007 | 317 (87.81%) | 172 (88.66%) | 145 (86.83%) | 0.596 | <0.001 |
| Diagnosis at ICU admission |  |  |  | 0.042 |  |  |  | 0.004 | 0.708 |
| Respiratory system | 147 (23.98%) | 126 (25.98%) | 21 (16.41%) |  | 93 (25.76%) | 63 (32.47%) | 30 (17.96%) |  |  |
| Circulatory system | 143 (23.33%) | 101 (20.82%) | 42 (32.81%) |  | 90 (24.93%) | 38 (19.59%) | 52 (31.14%) |  |  |
| Neoplasm | 102 (16.64%) | 80 (16.49%) | 22 (17.19%) |  | 52 (14.40%) | 32 (16.49%) | 20 (11.98%) |  |  |
| Digestive system | 44 (7.18%) | 37 (7.63%) | 7 (5.47%) |  | 21 (5.82%) | 7 (3.61%) | 14 (8.38%) |  |  |
| Infectious  (Including covid–19) | 44 (7.18%) | 37 (7.63%) | 7 (5.47%) |  | 32 (8.86%) | 18 (56.25%) | 14 (8.38%) |  |  |
| Others | 133 (21.7%) | 104 (21.44%) | 29 (22.66%) |  | 73 (20.22%) | 36 (18.56%) | 37 (22.16%) |  |  |
| Albumin (mg/dL) | 2.9 ± 0.9  (2.8, 2.4–3.2) | 2.8 ± 0.8  (2.7, 2.4–3.1) | 3.2 ± 1.1  (3.1, 2.7–3.5) | <0.001 | 2.7 ± 0.4  (2.7, 2.5–2.9) | 2.6 ± 0.4  (2.7, 2.4–2.9) | 2.8 ± 0.5  (2.8, 2.6–3.1) | <0.001 | 0.001 |
| CRP (mg/L) | 12.0 ± 9.3  (9.5, 4.3–18.9) | 12.8 ± 9.3  (10.8, 4.8–19.8) | 9.1 ± 8.5  (6.2, 2.9–14.1) | <0.001 | 8.9 ± 7.5  (6.4, 3.5–11.6) | 9.3 ± 7.6  (6.5, 4.0–11.9) | 8.3 ± 7.3  (6.3, 3.3–11.0) | 0.223 | <0.001 |
| Lactate (mg/dL) | 3.4 ± 4.2  (2.0, 1.3–3.4) | 3.7 ± 4.5  (2.1, 1.4–3.7) | 2.2 ± 2.3  (1.4, 1.1–2.2) | <0.001 | 2.2 ± 2.7  (1.5, 1.1–2.2) | 2.5 ± 3.4  (1.6, 1.1–2.7) | 1.7 ± 1.2  (1.3, 1.1–1.9) | 0.002 | <0.001 |
| WBC (/mm3) | 13.2 ± 10.6  (10.7, 7.7–15.8) | 13.7 ± 11.3  (11.3, 7.9–16.5), | 11.2 ± 7.3  (10.1, 7.2–12.6) | 0.002 | 11.9 ± 7.5  (10.3, 7.5–14.6) | 12.4 ± 8.4  (10.8, 7.7–16.3) | 11.4 ± 6.3  (10.0, 7.5–13.1) | 0.191 | 0.029 |
| Lymphocyte (%) | 9.8 ± 9.3  (7.7, 4.6–11.9) | 8.9 ± 8.4  (7.0, 4.4–10.7) | 13.0 ± 11.6  (10.6, 6.2–16.0) | <0.001 | 10.7 ± 9.8  (8.5, 4.8–13.3) | 9.8 ± 10.4  (7.0, 4.0–11.7) | 11.8 ± 8.9  (10.5, 5.5–15.5) | 0.044 | 0.145 |
| 28–day mortality |  |  |  | <0.001 |  |  |  | <0.001 | 0.489 |
| Death (N, %) | 153(24.96%) | 141(29.07%) | 12(9.38%) |  | 83(22.99%) | 66 (34.02%) | 17 (10.18%) |  |  |

* Calorie adequacy is calculated by dividing total calorie supply by calorie requirements.

Data were partially missing for albumin (1), CRP (17), lactate (52), lymphocyte (3) in acute phase and albumin (3), CRP (11), lactate (59), WBC (1), lymphocyte (3) in late phase, respectively. ICU intensive care unit; BMI body mass index; NPO Nothing per oral; EN Enteral Nutrition; PN Parenteral Nutrition; CRP C-reactive protein; WBC white blood cells.
